# Propargylglycine inhibits hypotaurine/taurine synthesis and elevates cystathionine and homocysteine concentrations in primary mouse hepatocytes

**DOI:** 10.1007/s00726-015-1948-7

**Published:** 2015-03-13

**Authors:** Halina Jurkowska, Martha H. Stipanuk, Lawrence L. Hirschberger, Heather B. Roman

**Affiliations:** 1 Division of Nutritional Sciences, Cornell University, Ithaca, NY 14853 USA; 2 Chair of Medical Biochemistry, Jagiellonian University Medical College, Kopernika 7 St., 31-034 Kraków, Poland

**Keywords:** Cysteine dioxygenase, Taurine, Hypotaurine, Cystathionine, Homocysteine, Cysteine desulfuration

## Abstract

Our investigation showed that hepatocytes isolated from cysteine dioxygenase knockout mice (Cdo1^−/−^) had lower levels of hypotaurine and taurine than *Cdo1* ^+*/*+^ hepatocytes. Interestingly, hypotaurine accumulates in cultured wild-type hepatocytes. dl-propargylglycine (PPG, inhibitor of cystathionine γ-lyase and H_2_S production) dramatically decreased both taurine and hypotaurine levels in wild-type hepatocytes compared to untreated cells. Addition of 2 mM PPG resulted in the decrease of the intracellular taurine levels: from 10.25 ± 5.00 observed in control, to 2.53 ± 0.68 nmol/mg protein (24 h of culture) and from 17.06 ± 9.40 to 2.43 ± 0.26 nmol/mg protein (control vs. PPG; 48 h). Addition of PPG reduced also intracellular hypotaurine levels: from 7.46 ± 3.55 to 0.31 ± 0.12 nmol/mg protein (control vs. PPG; 24 h) and from 4.54 ± 3.20 to 0.42 ± 0.11 nmol/mg protein (control vs. PPG; 48 h). The similar effects of PPG on hypotaurine and taurine levels were observed in culture medium. PPG blocked hypotaurine/taurine synthesis in wild-type hepatocytes, suggesting that it strongly inhibits cysteinesulfinate decarboxylase (pyridoxal 5′-phosphate-dependent enzyme) as well as cystathionine γ-lyase. In the presence of PPG, intracellular and medium cystathionine levels for both wild-type and *Cdo1* ^*−/−*^ cells were increased. Addition of homocysteine or methionine resulted in higher intracellular concentrations of homocysteine, which is a cosubstrate for cystathionine β-synthase (CBS). It seems that PPG increases CBS-mediated desulfhydration by enhancing homocysteine levels in hepatocytes. There were no overall effects of PPG or genotype on intracellular or medium glutathione levels.

## Introduction

**Fig. 1:**
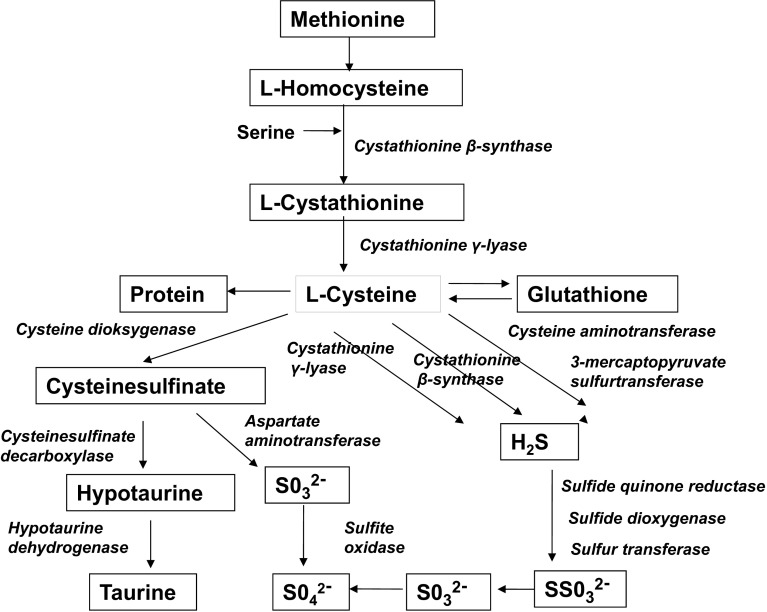
The production of taurine and desulfhydration pathways of cysteine

In mammals, cysteine is catabolized by several nonoxidative cysteinesulfinate-independent desulfuration pathways as well as by oxidative cysteinesulfinate-dependent pathways or it may be used for protein and glutathione synthesis (Fig. 1). Cysteine is catabolized by several desulfuration reactions that release sulfur in a reduced oxidation state, generating sulfane sulfur or hydrogen sulfide (H_2_S), which can be further oxidized to sulfate. Cysteine desulfuration is accomplished by alternate reactions catalyzed by cystathionine β-synthase (CBS), cystathionine γ-lyase (CTH), and 3-mercaptopyruvate sulfurtransferase (MPST) (Fig. 1). The oxidative pathway leads to production of taurine or sulfate (Stipanuk and Ueki 2011).

Taurine (2-aminoethanesulfonic acid) is synthesized endogenously from cysteine or via conversion from methionine, but also provided by diet, especially fish and seafood. The main pathway of taurine synthesis in mammals involves the oxidation of l-cysteine by the cysteine dioxygenase (CDO) to produce cysteine sulfinic acid. This acid is decarboxylated to hypotaurine by the cysteine sulfinic acid decarboxylase, which is finally oxidized to taurine by hypotaurine dehydrogenase. The conversion of the cysteinesulfinate to hypotaurine/taurine is minimized by the active transaminative catabolism of cysteinesulfinate to pyruvate and inorganic sulfur (Rosa et al. 2014; Stipanuk 2004; Hayes and Sturman 1981).

Taurine is the most abundant (~millimolar concentration) free amino acid that is involved in many fundamental biological functions (Imae et al. 2014; Hansen et al. 2006; Miyazaki and Matsuzaki 2014). In liver, the most established role of taurine is its conjugation with bile acids for excretion into bile (Danielsson 1963; Sjovall 1959). However, taurine has many other physiological and pharmacological roles in liver and other tissues, including stabilization of cellular plasma membrane (Pasantes et al. 1985), Ca^2+^ transport regulation (Huxtable 1992; Schaffer et al. 2010), osmoregulation (Nieminen et al. 1988; Huxtable 1992; Schaffer et al. 2010), antioxidant effects (Huxtable 1992; Nakamura et al. 1993; Schaffer et al. 2010; Rosa et al. 2014), anti-inflammation (Rosa et al. 2014), and detoxification (Huxtable 1992). Taurine supplementation has shown protective effects—such as antioxidant properties—to improve insulin sensitivity and dyslipidemia, as well as fatty liver disease, in rat models of obesity, diabetes type 2, and steatohepatitis (Deminice et al. 2014; Imae et al. 2014; Balkan et al. 2002; Rosa et al. 2014; Xiao et al. 2008).

In liver, taurine is abundantly maintained by endogenous biosynthesis and exogenous transport systems (Miyazaki and Matsuzaki 2014). Hepatic taurine synthesis is largely restricted by the low availability of cysteinesulfinate as substrate for cysteinesulfinate decarboxylase, and taurine production is increased when cysteinesulfinate increases in response to an increase in the hepatic cysteine concentration and the associated increase in cysteine dioxygenase activity (Stipanuk 2004).

Homocysteine and taurine are both sulfur-containing amino acids sharing the same biosynthetic pathway. As an end product of the transsulfuration pathway in homocysteine metabolism, taurine supplementation may play a crucial role in methionine metabolism in liver (Deminice et al. 2014).

In this study, determination of taurine and hypotaurine levels in the primary cultures of hepatocytes partially confirmed previous results from the experiments performed on mice (Roman et al. 2013; Ueki et al. 2011), and added new insights to our understanding of metabolism of the studied metabolites. Taurine level in *Cdo1* ^*−/−*^ hepatocytes is low, as was observed previously in the liver of *Cdo1* ^*−/−*^ mice. Taurine levels in the cultured hepatocytes isolated from Cdo1^−/−^ mice ranged from 4.6 to 6.2 % of the corresponding wild-type levels (this study), whereas taurine levels in the liver of Cdo1^−/−^ mice fed on taurine-free diets were found to be consistently less than 5 % of the wild-type levels (previously published study). Hypotaurine level is negligible in *Cdo1* ^*−/−*^ hepatocytes as in the liver of *Cdo1* ^*−/−*^ mice, reflecting the block in conversion of cysteine to cysteinesulfinate which is a precursor in the process of hypotaurine/taurine synthesis.

Interestingly, we have found in this study that significant differences in hypotaurine and taurine levels in wild-type hepatocytes compared to liver of wild-type mice were shown. We have shown that hypotaurine accumulates in cultured wild-type hepatocytes.

In the present study, we have also investigated the effect of propargylglycine (PPG; inhibitor of gamma-cystathionase) on hepatic hypotaurine and taurine synthesis and the levels of cystathionine, total unbound homocysteine, and glutathione in hepatocytes isolated from wild-type and Cdo1 knockout male mice. Interestingly, inhibitory effects of PPG on hypotaurine/taurine synthesis were found in wild-type hepatocytes. In addition, this study proved that PPG, as well as homocysteine and methionine, increase cystathionine and homocysteine levels in the cells and in the conditional medium, which might result in an intensification of desulfuration processes.

## Materials and methods

### Primary hepatocytes and cell culture

Cdo1-null (*Cdo1* ^−*/*−^ *)* and wild-type (*Cdo1* ^+*/*+^ *)* mice for this study were generated by crossing C57BL/6 *Cdo1* ^+*/*−^ male and female mice as described previously (Jurkowska et al. 2014; Roman et al. 2013; Ueki et al. 2011). All experimental procedures involving live animals were conducted with the approval of the Cornell University Institutional Animal Care and Use Committee (#2009-0138). Mice (42–100 days of age) were euthanized with an overdose of isoflurane, and hepatocytes were quickly isolated by Dominy’s method (Dominy et al. 2012) with some modifications as described by Jurkowska et al. (2014). Viability of isolated hepatocytes was determined by trypan blue exclusion and was routinely greater than 90 %.

Isolated hepatocytes were plated on collagen-coated 6-well plates (4 × 10^4^ cells per cm^2^) in DMEM (Gibco #12800-017, containing 1 mM pyruvate, 25 mM glucose, and 4 mM glutamine) supplemented with 10 % (vol/vol) fetal bovine serum (FBS), 100 units/ml penicillin, 100 µg/ml streptomycin, 1 µM dexamethasone, 0.1 µM insulin, and an additional 1 mM sodium pyruvate and incubated at 37 °C in an atmosphere of 5 % CO_2_. At 2 h after plating, the plating medium was replaced with fresh culture medium consisting of DMEM supplemented with 1 mM sodium pyruvate, 100 units/ml penicillin, 100 µg/ml streptomycin, 0.2 % (vol/vol) of bovine serum albumin fraction V (instead of FBS), 100 nM dexamethasone, and 1 nM insulin (Sigma–Aldrich #I9278). After 24 h, culture medium was replaced with treatment medium (total volume of 3.0 ml per well).

All treatment media contained 0.7 mM cyst(e)ine and 0.05 mM bathocuproine disulfonic acid (BCS, Sigma–Aldrich). 2 mM dl-propargylglycine (PPG, Sigma–Aldrich), 0.2 mM l-homocysteine (Hcy, Sigma–Aldrich), or 0.5 mM l-methionine (Met, Research Products International, Inc) was added as indicated.

Hepatocytes were cultured in the treatment medium for 24 and 48 h prior to harvest. For measurement of metabolite levels in the medium, the medium was removed prior to harvesting the hepatocytes and immediately frozen and stored at −80 °C for later analysis. For measurement of metabolite levels in cells, hepatocytes were harvested by treating with 0.25 % trypsin (Gibco #25200), washing with ice-cold PBS to remove trypsin, suspending released hepatocytes in ice-cold PBS, and centrifuging the cell suspension at 1600×*g* for 10 min at 4 °C to obtain the pelleted cells, which were immediately frozen and stored at −80 °C for later analysis.

### Measurement of hypotaurine, taurine, cystathionine, and total unbound homocysteine and glutathione

The homogenates and media were prepared as described previously (Jurkowska et al. 2014). Frozen hepatocytes were thawed on ice and resuspended in 0.1 M phosphate buffer, pH 7.5, containing 2 mM tris(2-carboxyethyl)phosphine (TCEP) to reduce disulfide bonds linking thiols to protein sulfhydryl groups or to each other. The mixture was then sonicated for three 5 s intervals at 4 °C, and the homogenates were used for HPLC determinations and for protein level determination. Frozen samples of culture medium were thawed on ice, and 2 µl of 200 mM TCEP (in 125 mM borate buffer, pH 9.0) was added to 200 µl of medium to yield a final concentration of 2 mM TCEP for reducing disulfide bonds.

For the measurement of hypotaurine, taurine, and cystathionine, the prepared homogenate or medium was mixed with one volume of 5 % (wt/vol) sulfosalicylic acid, and the mixture was centrifuged at 15,000×*g* for 15 min at 4 °C to obtain the acid supernatant. Taurine and hypotaurine were measured by HPLC as described previously (Ueki et al. 2012). Samples were derivatized with *o*-phthaldialdehyde (OPA) and separated on a C18 column by gradient elution using 0.05 M potassium phosphate buffer (pH 7.0) −3.5 % (vol/vol) tetrahydrofuran mobile phase without or with 40 % (vol/vol) acetonitrile. Detection of OPA-derivatized compounds was performed using excitation and emission peaks at 360 and 455 nm, respectively.

For the determination of total unbound glutathione and homocysteine, reduced homogenate or medium was mixed with an equal volume of 5 % (wt/vol) trichloroacetic acid, and the mixture was centrifuged for 15 min at 15,000×*g* at 4 °C to obtain the acid supernatant. Total glutathione and homocysteine were measured by HPLC as described previously (Ueki et al. 2012). Briefly, thiols were derivatized with 7-fluorobenzo-2-oxa-1,3-diazole-4-sulphonate, and chromatography was carried out on a C18 reversed-phase column with a mobile phase of 0.1 M potassium phosphate buffer (pH 2.1) with 5 % (vol/vol) acetonitrile. Fluorescence of derivatives in the eluate was detected using an excitation wavelength of 385 nm and an emission wavelength of 515 nm.

### Protein assay

The protein level of cell homogenates was determined by the BCA Protein Assay Kit (Thermo Scientific/Pierce) using bovine serum albumin (BSA) as a standard.

### Statistical analysis

Results were analyzed as a full factorial least squares model using JMP version 10 (SAS, Cary, NC). Post hoc individual pairwise comparisons of least squares means in the model were made using Tukey’s comparisons; comparisons were considered significant at *p* < 0.05. Values for hypotaurine, taurine, homocysteine, and glutathione were log or square root transformed prior to statistical analysis.

## Results

In this study, the experiments were performed on hepatocytes isolated from wild-type and Cdo1 knockout male mice.

### Comparison of oxidative metabolism of cysteine in hepatocytes isolated from wild-type and Cdo1 knockout mice

**Fig. 2:**
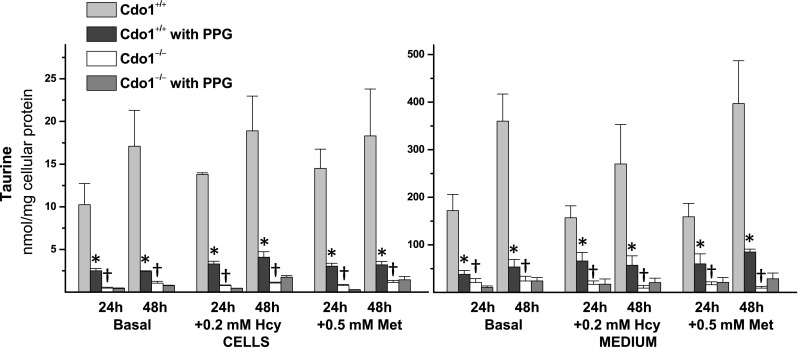
Effect of 2 mM dl-propargylglycine (PPG) on taurine levels in hepatocytes and in the culture medium for preparations of hepatocytes from *Cdo1* ^+*/*+^ and *Cdo1* ^*−/−*^ mice that were cultured in basal [0.7 mM cyst(e)ine] medium or basal medium supplemented with 0.2 mM l-homocysteine (Hcy) or with 0.5 mM l-methionine (Met) for either 24 or 48 h. Results are the mean ± SEM from three independent experiments. Statistical analysis was done using a standard least squares model followed by Tukey’s mean comparison procedure; significance was accepted at *p* < 0.05. Data were log (intracellular) or square root (medium) transformed before statistical analysis. * = +PPG value is different than the corresponding −PPG value; † = Cdo1^−/−^ value is different than the corresponding Cdo1^+/+^ value

**Fig. 3:**
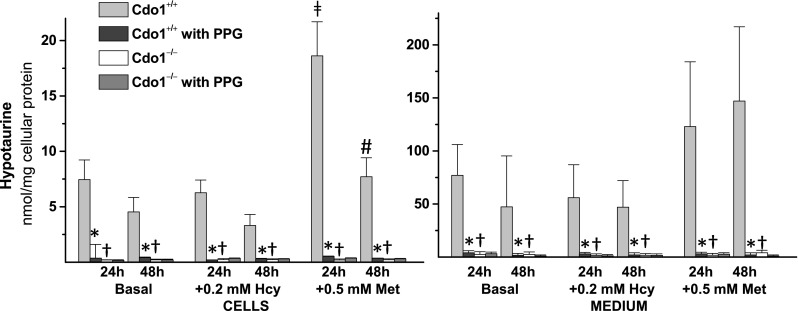
Effect of 2 mM dl-propargylglycine (PPG) on hypotaurine levels in hepatocytes and in the culture medium for preparations of hepatocytes from male *Cdo1* ^+*/*+^ and *Cdo1* ^*−/−*^ mice that were cultured in basal [0.7 mM cyst(e)ine] medium or basal medium supplemented with 0.2 mM l-homocysteine (Hcy) or with 0.5 mM l-methionine (Met) for either 24 or 48 h. Results are the mean ± SEM from three independent experiments. Statistical analysis was done using a standard least squares model followed by Tukey’s mean comparison procedure; significance was accepted at *p* < 0.05. Data were square root transformed before statistical analysis. * = +PPG value is different than the corresponding −PPG value; † = Cdo1^−/−^ value is different than the corresponding Cdo1^+/+^ value

Taurine and hypotaurine levels in cells and medium are reported in Figs. 2 and 3. Taurine levels ranged from 10 to 17 nmol/mg protein in hepatocytes from *Cdo1* ^+*/*+^ mice (Fig. 2), whereas hypotaurine levels ranged from 4 to 8 nmol/mg protein in hepatocytes from wild-type mice (Fig. 3). Both taurine and hypotaurine levels remained very low in *Cdo1* ^*−/−*^ hepatocytes and in their culture medium compared to *Cdo1* ^+*/*+^ hepatocytes (Figs. 2, 3). Taurine levels ranged from 4.6 to 6.2 % of wild-type levels in hepatocytes from *Cdo1* ^*−/−*^ mice (Fig. 2), whereas hypotaurine levels were about 0.5 % of wild-type levels in hepatocytes from *Cdo1* ^*−/−*^ mice (Fig. 3).

### Effect of propargylglycine on oxidative metabolism of cysteine in hepatocytes from wild-type mice

Addition of 2 mM PPG resulted in a dramatic reduction of both cellular and medium taurine levels for wild-type hepatocytes, with intracellular taurine levels being about 20 % of the corresponding non-PPG levels and medium taurine levels being about 25 % of the corresponding non-PPG levels (Fig. 2). Similarly, addition of 2 mM PPG reduced cellular and medium hypotaurine levels for wild-type hepatocytes, with intracellular hypotaurine levels being 3–9 % of the corresponding non-PPG levels and medium hypotaurine levels being 1–5 % of the corresponding non-PPG levels (Fig. 3). There was little effect of 0.5 mM Met or 0.2 mM Hcy addition on intracellular or medium taurine or hypotaurine levels, except for significantly higher hypotaurine levels in wild-type cells cultured in +Met/No PPG medium (Figs. 2, 3).

### Effect of propargylglycine on cystathionine, homocysteine, and total glutathione levels in hepatocytes isolated from wild-type and Cdo1 knockout mice

**Fig. 4:**
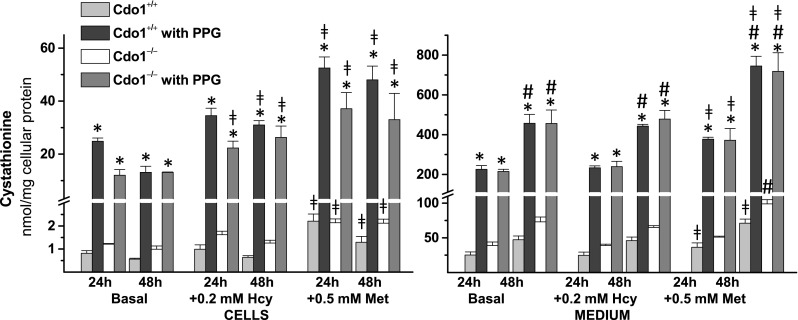
Effect of 2 mM dl-propargylglycine (PPG) on cystathionine levels in hepatocytes and the culture medium for preparations of hepatocytes from *Cdo1* ^+*/*+^ and *Cdo1* ^*−/−*^ mice that were cultured in basal [0.7 mM cyst(e)ine] medium or basal medium supplemented with 0.2 mM l-homocysteine (Hcy) or with 0.5 mM l-methionine (Met) for either 24 or 48 h. Results are the mean ± SEM from three independent experiments. Statistical analysis was done using a standard least squares model followed by Tukey’s mean comparison procedure; significance was accepted at *p* < 0.05. Data were log transformed before statistical analysis. * = + PPG value is different than the corresponding −PPG value; # = 48 h value is different than the corresponding 24 h value; ╪ = +Hcy or +Met value is different than the corresponding basal value

Addition of PPG increased both intracellular and medium cystathionine levels for both wild-type and *Cdo1* ^*−/−*^ cells and for all culture conditions (Fig. 4). Intracellular cystathionine levels in wild-type hepatocytes cultured in the presence of PPG were 34-times those of the corresponding non-PPG values, whereas those for *Cdo1* ^*−/−*^ hepatocytes cultured in the presence of PPG were 14-times those of the corresponding non-PPG values, with the lower fold increases in *Cdo1* ^*−/−*^ cells being related to their higher basal levels of cystathionine. Medium cystathionine levels in hepatocytes treated with PPG were an average of 7.5- and 6.6-times those of the non-PPG control values for wild-type and *Cdo1* ^*−/−*^ hepatocytes, respectively. Addition of Met increased intracellular cystathionine levels to an average of 2.2-times and increased medium cystathionine levels to an average of 1.4-times those for cells cultured in basal medium. Addition of Hcy had no effect on intracellular or medium cystathionine levels in cells cultured in the absence of PPG, but did significantly increase intracellular cystathionine in cells cultured with PPG (Fig. 4).

**Fig. 5:**
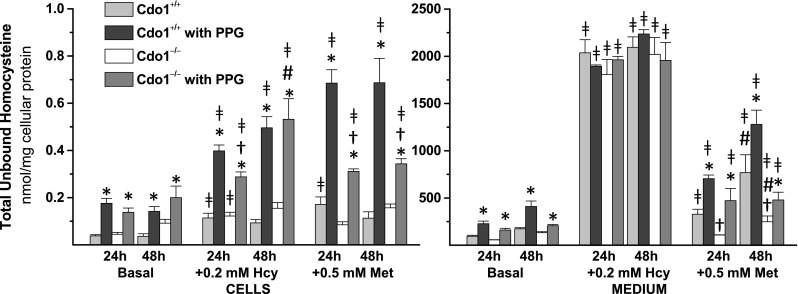
Effect of 2 mM dl-propargylglycine (PPG) on total unbound homocysteine levels in hepatocytes and in the culture medium for preparations of hepatocytes from *Cdo1* ^+*/*+^ and *Cdo1* ^*−/−*^ mice that were cultured in basal [0.7 mM cyst(e)ine] medium or basal medium supplemented with 0.2 mM l-homocysteine (Hcy) or with 0.5 mM l-methionine (Met) for either 24 or 48 h. Results are the mean ± SEM from three independent experiments. Statistical analysis was done using a standard least squares model followed by Tukey’s mean comparison procedure; significance was accepted at *p* < 0.05. Data were square root transformed before statistical analysis. * = + PPG value is different than the corresponding −PPG value; † = Cdo1^−/−^ value is different than the corresponding Cdo1^+/+^ value; # = 48 h value is different than the corresponding 24 h value; ╪ = +Hcy or +Met value is different than the corresponding basal value

Homocysteine, which along with serine or cysteine is a precursor of cystathionine, was elevated in hepatocytes treated with 2 mM PPG (Fig. 5). Intracellular total unbound homocysteine levels in cells treated with PPG were an average of 3.7-times those in corresponding cells not treated with PPG. Levels of homocysteine in the culture medium were also significantly elevated (2.4-times non-PPG control values) for hepatocytes cultured in the presence of PPG except for those cultured in medium with added Hcy. The intracellular level of total unbound homocysteine increased over time in cells cultured in +Hcy +PPG medium, presumably due to uptake from the medium; small increases in medium homocysteine were observed over time in cells cultured in +Met medium. The intracellular level of total unbound homocysteine was lower for *Cdo1* ^*−/−*^ than for wild-type cells when hepatocytes were cultured in +Met +PPG medium, probably reflecting the block in transsulfuration. Addition of Hcy or Met to the culture medium resulted in increase of intracellular homocysteine levels on average by 3.3- or 3.5-times, respectively, for cells cultured in basal medium. Levels of homocysteine in the medium were also affected by Hcy or Met addition. Addition of Hcy to the medium dramatically increased medium homocysteine levels, whereas addition of Met to the medium resulted in an elevation of homocysteine levels to 3.8-times basal levels for wild-type hepatocytes and 1.8-times basal levels for *Cdo1* ^*−/−*^ hepatocytes.

**Fig. 6:**
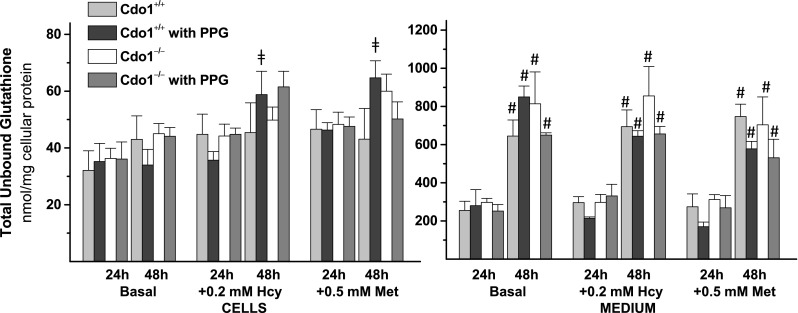
Effect of 2 mM dl-propargylglycine (PPG) on total unbound glutathione levels in hepatocytes and in the culture medium for preparations of hepatocytes from *Cdo1* ^+*/*+^ and *Cdo1* ^*−/−*^ mice that were cultured in basal [0.7 mM cyst(e)ine] medium or basal medium supplemented with 0.2 mM l-homocysteine (Hcy) or with 0.5 mM l-methionine (Met) for either 24 or 48 h. Results are the mean ± SEM from three independent experiments. Statistical analysis was done using a standard least squares model followed by Tukey’s mean comparison procedure; significance was accepted at *p* < 0.05. Data for medium glutathionine levels were square root transformed before statistical analysis. # = 48 h value is different than the corresponding 24 h value; ╪ = + Hcy or +Met value is different than the corresponding basal value

Total unbound glutathione levels are shown in Fig. 6. There were no overall effects of PPG or genotype on intracellular or medium glutathione levels. Medium total unbound glutathione levels, but not intracellular levels, significantly increased between 24 and 48 h of culture. Hcy or Met addition significantly increased intracellular glutathione levels only in wild-type hepatocytes cultured in the presence of PPG.

## Discussion

### Hypotaurine accumulates in cultured wild-type hepatocytes

Despite the similarly low levels of hypotaurine and taurine in cultured *Cdo1* ^*−/−*^ hepatocytes and in liver of *Cdo1* ^*−/−*^ mice, there were some notable differences in hypotaurine and taurine levels in wild-type hepatocytes compared to liver of wild-type mice. Interestingly, taurine levels in cultured wild-type hepatocytes were substantially lower than those measured in liver of wild-type mice, being 10–17 nmol/mg protein in hepatocytes cultured in basal medium (Fig. 2) compared to 55–80 nmol/mg protein in liver (Roman et al. 2013; Ueki et al. 2011). The lower taurine levels in cultured hepatocytes suggest that the whole animal may have a greater capacity for taurine conservation or a greater capacity for taurine synthesis than do isolated hepatocytes. A limited capacity of primary hepatocytes to oxidize hypotaurine to taurine may contribute to this difference because, in contrast to taurine levels, hypotaurine levels in cultured wild-type hepatocytes were higher than those in liver of wild-type mice, being 4–8 nmol/mg protein in cells cultured in basal medium (Fig. 3) compared to less than 0.8 nmol/mg protein in liver of wild-type mice. It is possible that other tissues or factors in the whole animal may be necessary for efficient oxidation of hypotaurine to taurine. The observation of hypotaurine accumulation in cultured hepatocytes was unexpected but not without precedent. In most tissues, hypotaurine levels are very low compared to taurine levels, and hypotaurine is virtually undetectable in mouse or rat plasma (Hirschberger et al. 1985; Nakamura et al. 2006; Roman et al. 2013). However, very high levels of hypotaurine have long been known to be present in male reproductive tissues (i.e., epididymal fluid, seminal plasma and sperm) (Holmes et al. 1992; Johnson et al. 1972), and we recently reported very high levels of hypotaurine in mouse pancreas (Roman et al. 2013). The accumulation of hypotaurine in cultured hepatocytes follows the report by Vitvitsky et al. (2011) of hypotaurine accumulation in primary astrocytes and neurons when these cells were incubated with cysteamine, cysteinesulfinate, or cysteine. The differential accumulation of hypotaurine in different cell types strongly suggests that hypotaurine conversion to taurine is regulated. In addition, the accumulation of hypotaurine in the culture medium as well as in the hepatocytes themselves, despite a cell culture environment with 20 % O_2_, suggests that hypotaurine oxidation is enzymatically catalyzed. Although earlier efforts to purify and characterize a putative hypotaurine dehydrogenase were not successful in identifying a protein whose activity was sufficient to account for the rates of hypotaurine oxidation observed in vivo (Huxtable 1989; Kontra and Oja 1980; Oja and Kontro 1981; Sumizu 1962; Stipanuk 1986), the accumulation of intra- and extra-cellular hypotaurine in cell culture studies, as well as the very high levels of hypotaurine in certain tissues of whole animals, raises new questions about the mechanism and the location of hypotaurine oxidation in the body.

### PPG blocks taurine synthesis by the cysteinesulfinate pathway

**Fig. 7:**
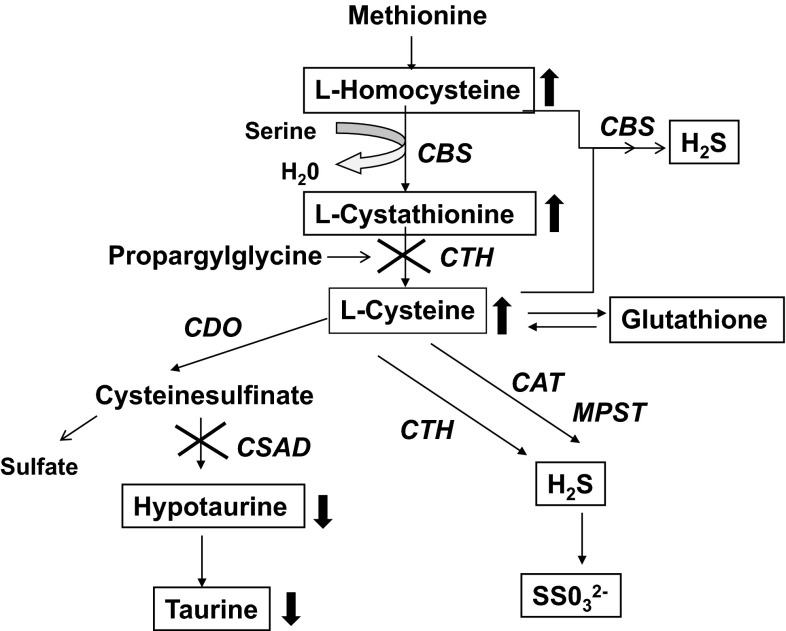
Effect of propargylglycine on hypotaurine/taurine synthesis and desulfhydration pathways in primary wild-type hepatocytes. *CDO* cysteine dioksygenase, *CSAD* cysteinesulfinate decarboxylase, *CTH* cystathionine γ-lyase, *CBS* cystathionine β-synthase, *CAT* cysteine aminotransferase, *MPST* 3-mercaptopyruvate sulfurtransferase

An unexpected finding was the dramatic effect of PPG on taurine synthesis in wild-type cells. PPG dramatically reduced hypotaurine and taurine levels in wild-type hepatocytes (Figs. 2, 3). The inhibition of hypotaurine and taurine production by PPG in wild-type cells, along with the low levels of hypotaurine and taurine in *Cdo1* ^*−/−*^ cells, suggests that PPG is an effective inhibitor of pyridoxal 5′-phosphate-dependent cysteinesulfinate decarboxylase, the enzyme that converts cysteinesulfinate formed from cysteine by CDO to hypotaurine. This finding implies that use of PPG to inhibit CTH activity will simultaneously block the conversion of cysteinesulfinate to hypotaurine/taurine (Fig. 7). Although a loss of cysteinesulfinate decarboxylase activity should not affect our assessment of cysteine desulfhydration in hepatocytes, it would affect end points involving taurine or hypotaurine determinations.

### PPG increases CBS-mediated desulfhydration by enhancing homocysteine levels in hepatocytes

Genotype did not have a consistent effect on intracellular homocysteine levels (Fig. 5), similarly to our previous observations for liver of *Cdo1* ^*−*/−^ and wild-type mice (Roman et al. 2013). Hcy and Met were added in an effort to increase cosubstrate for CBS-catalyzed desulfhydration and to increase levels of S-adenosylmethionine as an activator of CBS (Singh et al. 2009; Finkelstein et al. 1975). Thus, addition of Hcy or Met resulted in higher intracellular concentrations of homocysteine (Fig. 5), which is a cosubstrate for CBS-catalyzed transsulfuration (Ser + Hcy → Cystathionine + H_2_O) and for CBS-catalyzed cysteine desulfhydration (Cys + Hcy → Cystathionine + H_2_S). Consequently, addition of Met results in increasing the intracellular and medium cystathionine levels (Fig. 4). This confirms our earlier results that accumulation of cystathionine in Cdo1-null hepatocytes compared to wild-type hepatocytes supports an important role of CBS in cysteine desulfhydration in liver (Jurkowska et al. 2014).

PPG treatment of wild-type hepatocytes increased intracellular homocysteine (Fig. 5) and cystathionine (Fig. 4) levels. Also, we previously showed that PPG increased cysteine levels in hepatocytes from wild-type mice (Jurkowska et al. 2014). This seems to suggest that PPG, by raising cystathionine and homocysteine levels in the face of a block in CTH activity, might increase CBS-mediated desulfhydration (Fig. 7).

Total unbound glutathione levels were not affected by PPG treatment in cultured hepatocytes (Fig. 6). As was reported previously, hepatic GSH concentration was also not decreased by the addition of PPG to the various diets in rats (Cresenzi et al. 2003). This may be explained by the observation that hepatic GSH concentration remains relatively constant when cysteine concentration is >0.04 µmol/g (Stipanuk et al. 2002) and also by the higher priority of GSH synthesis than cysteine catabolic pathways for cysteine when its supply is limited (Stipanuk et al. 1992).
